# Low-Energy Photoelectron Spectroscopy and Scattering from Aqueous Solutions and the Role of Solute Surface Activity

**DOI:** 10.1021/jacs.5c04263

**Published:** 2025-06-02

**Authors:** Stephan Thürmer, Dominik Stemer, Florian Trinter, Igor Yu Kiyan, Bernd Winter, Iain Wilkinson

**Affiliations:** † Department of Chemistry, Graduate School of Science, Kyoto University, Kitashirakawa-Oiwakecho, Sakyo, 606-8501 Kyoto, Japan; ‡ Molecular Physics Department, 28259 Fritz-Haber-Institut der Max-Planck-Gesellschaft, Faradayweg 4-6, 14195 Berlin, Germany; § Institut für Kernphysik, Goethe-Universität Frankfurt, Max-von-Laue-Straße 1, 60438 Frankfurt am Main, Germany; ∥ Institute for Electronic Structure Dynamics, 28340 Helmholtz-Zentrum Berlin für Materialien und Energie, Hahn-Meitner-Platz 1, 14109 Berlin, Germany

## Abstract

Experimental insights into low-kinetic-energy electron scattering in aqueous solutions are essential for an improved understanding of electron-driven chemistry and radiobiology, and the development and informed application of aqueous-phase electron-based spectroscopy and dichroism methods. Generally, in aqueous environments and for electron kinetic energies below 12–15 eV, significant and, thus far, incompletely understood low-energy-transfer inelastic electron scattering with solvent molecules preponderates. This leads to cascades of tens-of-meV kinetic-energy losses that distort nascent photoelectron spectra, prevent direct and accurate electron-binding-energy measurements, and limit possibilities to determine electron-scattering cross sections at especially low electron kinetic energies. Here, we quantify aqueous-phase inelastic-scattering-based energy losses using 1–30 eV kinetic energy photoelectrons and liquid-jet photoemission spectroscopy, specifically by photoionizing an exemplary surface-active solute and comparing the results with those from the homogeneously distributed aqueous solvent. Thereby, we identify a general ≳17 eV electron-kinetic-energy requirement for the direct and accurate measurement of aqueous-phase electron binding energies, irrespective of interfacial concentration profiles. Further, at electron kinetic energies from 10 eV down to a few-eV above the ionization threshold, we observe and quantify lower degrees of scattering for photoelectrons generated from surface-active solutes, allowing moderately distorted surface-active-solute photoemission peaks to be resolved down to just few-eV electron kinetic energies. These results demonstrate that liquid-jet photoemission spectroscopy can be used to probe interfacial surface-active-solute dynamics and dichroism effects close to ionization thresholds, in stark contrast to similar experiments on homogeneously distributed solution components. Furthermore, they offer novel insights into low-electron-kinetic-energy scattering in aqueous environments, thereby addressing the current lack of reliable experimental data in this critical energy range.

## Graphical abstract

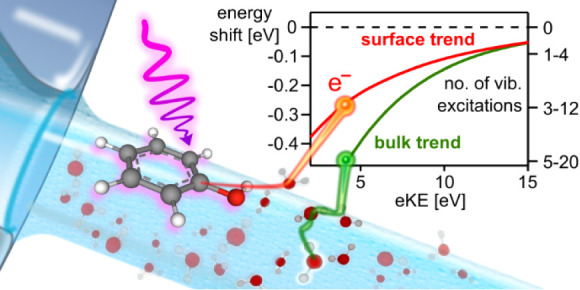

## Introduction

Differential valence-electron energetics in aqueous solutions provide the driving forces for a broad range of (bio)­chemical and environmental processes. The accurate measurement of electron binding energies (BEs) of solutes and solvents – which reveal such electron energetics and are directly accessed using liquid-jet photoemission spectroscopy (LJ-PES)^1,2^ – is correspondingly crucial. However, photoelectrons (PEs) inevitably scatter in a condensed-phase environment, leading to detrimental background signals and the potential for erroneous BE determinations, especially at low PE kinetic energies (KEs), where electron-scattering mechanisms and cross-sections continue to be debated.^3–7^ In the case of liquid water and spatially homogeneous aqueous solutions, we found that PE features are increasingly distorted, diminished in intensity, and difficult to separate from the scattered-electron background for electron KEs lower than 12–15 eV (∼14 eV).^8^ This is the energetic crossover region where electronically inelastic scattering (ionization, excitation, and dissociation) becomes improbable and vibrational scattering dominates, below which PE-peak distortions predominantly arise from the latter. At such low KEs, cascades of tens-of-meV, vibrationally or translationally inelastic KE losses occur that are small enough to produce significant signal backgrounds directly beneath the broad PE peaks encountered in LJ-PES experiments;^8–10^ we term such scattering quasi-elastic due to the inseparability of the scattered electrons from the as-generated photoelectron signals and to differentiate them from electronically scattered electrons and associated resolvable multiple-eV energy losses. Ultimately, the low-energy quasi-elastic scattering processes lead to an essentially complete loss of PE peak structure at electron KEs of ≲5 eV,^1^ marking a low-KE limit (LKEL) for the isolation of *highly distorted* PE peaks from homogeneously distributed aqueous solutes or the water solvent. Importantly, significantly higher electron KEs of 15–19 eV (∼17 eV) have been shown to be required to directly and accurately measure aqueous-phase BEs.^1^ ^,^ [Fn fn1] Therefore, care must be taken when performing LJ-PES experiments with low-photon-energy sources; considering the lowest BEs of common aqueous solutes (often ≳8 eV) and liquid water (neat water: 11.33 ± 0.03 eV^1^), ≳25 eV and ≳28 eV photon energies are respectively and generally required to enable direct and accurate BE measurements,[Fn fn2] i.e., higher single-photon energies than those generally afforded in optical laser laboratories or by commonly implemented He Iα discharge light sources, or the cumulative multiphoton excitation energies than can be implemented with optical lasers at appropriate intensities to study liquid-phase samples.

In contrast to PEs originating in the aqueous bulk, PEs from interfacial species will, on average, travel a less significant distance in a liquid-phase environment and are expected to experience far fewer scattering events before they can escape into a vacuum. Hence, surface-active solute PE features are likely detectable at lower KEs and on reduced local electron-scattering backgrounds in LJ-PES experiments. Although often not explicitly acknowledged, this has enabled LJ-PES studies on surface-active species using total excitation, i.e., ionizing, laser-photon energies below 10 eV; see, e.g., refs. 11–15 A related major motivation for the present work is the explicit measurement of reduced LKELs for PE peak detection and energetic thresholds to directly and accurately determine BEs from surface-active aqueous solutes. To achieve this, we measured LJ-PES spectra from exemplary aqueous solutions of surface-active phenol, PhOH_(aq)_. Such solutions have been well studied using UV LJ-PES and total excitation energies as low as 9 eV (leading to PE KEs < 1 eV).^12–14,16^ Furthermore, valence and core-level ionization studies have been performed using soft-X-ray synchrotron-radiation facilities, where the PE KEs were in the hundred-eV range,^17,18^ i.e., well beyond any previously considered LKEL values and thresholds for direct and accurate BE determinations.

Here, we compare valence PE spectra from 50 mM PhOH_(aq)_ solutions with the corresponding spectra from the liquid water solvent and valence and carbon 1s core-level X-ray PE spectra from the same 50 mM PhOH_(aq)_ solutions. In these experiments, the photon energies are suitably varied such that the PEs from the phenol and water lowest-BE, valence ionization channels and phenol core-level ionization channels have KEs ranging from near-zero to approximately 30 eV, i.e., spanning the bulk-aqueous-solution LKEL and threshold for accurate BE measurements. As in the case of homogeneously distributed species in aqueous solutions,^8^ the surface-active PhOH_(aq)_ LKEL and accurate-BE-measurement thresholds are found to be equivalent for valence- and core-level ionization. Notably, and rather surprisingly, similar ∼17 eV accurate-BE-measurement thresholds are determined for both aqueous-phase surface-active solutes and homogeneously distributed solution components. However, at electron KEs < 10 eV, the magnitudes of the scattering-based PE shifts and peak distortions for the surface-active solutes are found to be significantly lower than those observed for homogeneously distributed aqueous solutes and liquid water. This implies that the surface-active-solute LKEL shifts to lower energies, allowing isolated, yet somewhat distorted, PE features to be extracted at lower photon and, hence, kinetic energies in surface-active-solute LJ-PES measurements.

## Methods

Phenol of 99% purity (Sigma-Aldrich) was added to highly demineralized water (conductivity ∼ 0.2 μS/cm) to achieve a 50 mM bulk concentration. No pH adjustment was performed, which yields an acidic solution with a natural pH of ∼5. This pH value is far away from the p*K*_*a*_ value of 10 in the bulk and 11.7 at the air–water interface, beyond which phenol deprotonates to form phenolate.^18,19^ Thus, any signal contribution of phenolate is expected and observed to be negligible. NaCl was also added to the sample solutions at 50 mM concentrations to ensure sufficient liquid jet (LJ) electrical conductivity for LJ-PES measurements, mitigating potentially deleterious sample charging effects^20^ and enabling LJ biasing and spectral cutoff energy referencing.^1^ While NaCl has the same average concentration as the phenol solute, the surface activity of PhOH_(aq)_ yields an orders-of-magnitude higher concentration at the probed interface;^18^ NaCl is effectively undetectable in these experiments, and the associated Na^+^_(aq)_ and Cl^–^_(aq)_ solute PE features can correspondingly be neglected. Based on the surface-tension and LJ-PES results reported in ref 18 under the solution conditions adopted here, the PhOH_(aq)_ surface concentration and fraction are respectively estimated to be 1.2–1.8 × 10^14^ molecules/cm^2^ and 0.33–0.42. We note that interfacial PhOH_(aq)_ solute–solute interactions are expected at such solute concentrations,^21^ leading to lower BEs compared to those observed at the low-solute-concentration limit.^18^ However, these interfacial agglomeration phenomena and associated concentration-dependent BEs have little bearing on the general electron-scattering phenomena explored and discussed here, particularly given that all measurements were performed with the same 50 mM PhOH_(aq)_ solutions.

The sample solutions were introduced into the LJ-PES measurement chambers as cylindrical LJs, as formed using glass-capillary nozzles of 20–35 μm inner diameter. 0.6–0.8 mL/min sample-injection flow rates were maintained with a Techlab 2/ED (PhOH_(aq)_) high-performance liquid chromatography (HPLC), a Teledyne ISCO 500 D (H_2_O_(l)_) syringe pump in the EUV-ionization experiments, and a Shimadzu LC-20AD HPLC pump equipped with a degasser (Shimadzu DGU-20A5R) in the soft-X-ray-ionization experiments. The LJ assemblies featured metallic inserts and water-cooled jackets for electrical bias and thermal control, respectively; the LJ bias potentials were set between −20 V and −64 V to expose the LET and spectral cutoff, and the temperature was stabilized at 8 or 10 °C using LJ chiller units to reduce the sample vapor pressures. The ionizing-radiation–LJ interaction points were situated ∼1 mm downstream from the nozzles, where somewhat lower sample temperatures are expected due to evaporative cooling.

The EUV valence-band LJ-PES spectra were recorded in the Ultrafast Laser Laboratory for Applied Sciences (ULLAS) facility at the Helmholtz-Zentrum Berlin für Materialien und Energie (HZB). The implemented EUV photon energies were produced via high-order harmonic generation (HHG) of the output of a titanium:sapphire laser system, which delivered horizontally polarized, 1 mJ, 25 fs-duration (FWHM), 800 nm-central-wavelength pulses at a repetition rate of 5 kHz to an EUV source and beamline. These pulses were focused into an Ar-gas-filled cell (∼40 mbar fill, 2 mm cell length) to produce a frequency comb of odd-order harmonics over a spectral range of 4.5 to 45 eV. Individual harmonics (7th, 9th, 11th, 15th, 17th, 21st, and 25th) were selected using an upgraded version of a previously described reflection-zone-plate EUV monochromator system^22^ to achieve relatively broad 170–400 meV EUV bandwidths.[Fn fn3] The selected high harmonics were subsequently relay-imaged onto the LJ samples using a toroidal mirror, resulting in ∼60 μm (1/e^2^) EUV beam diameters at the LJ. The on-target photon fluxes were attenuated to ∼10^10^ photons/s for these measurements to avoid deleterious ionization-induced charging effects. The LJ-PES signals were recorded using a commercial, differentially pumped THEMIS 600-EP (SPECS GmbH) angle-resolved time-of-flight (ToF) PE spectrometer system, which was operated in its field-free mode with a −20 or −25 V LJ bias, a grounded spectrometer entrance aperture, and over an electron KE range of 15 to 55 eV, where a 20 to 70 meV spectrometer KE resolution was achieved. This yielded total experimental energy resolutions of the order of 170–410 meV. The spectrometer ToF axis was aligned parallel to the EUV-beam polarization, and the LJ was introduced to the chamber orthogonally to both the EUV-propagation and electron-detection axes in all the valence-ionization experiments. During the LJ experiments, the LJ was positioned ∼0.5 mm away from the 0.5 mm-diameter entrance aperture to the ToF spectrometer. The average pressure inside the interaction chamber was kept below 4 × 10^–4^ mbar by a combination of a turbomolecular pump (TMP, 1450 L/s pumping speed for N_2_ gas) and two liquid-nitrogen-cooled traps with total pumping speeds of ∼45000 L/s for water. The electron-detection chamber was maintained below 2 × 10^–6^ mbar using three additional TMPs on the ToF spectrometer. The LJ was frozen out and collected by one of the cold traps ∼30 cm below the LJ injection point. The HHG photon energies and photoemission-spectrometer performance were calibrated using single-photon ionization of nitric oxide, xenon, or argon gas, as delivered to the EUV interaction region as neat atomic/molecular gas jets using a 50 μm-diameter pinhole nozzle and 400–900 mbar stagnation pressures. The LJ-PES valence-band spectra were calibrated using the predetermined photon energies, known higher-EUV-photon-energy liquid-water 1b_1_ BEs,^1^ and the solution cutoff features in the liquid-phase spectra, where the latter define the true zero-kinetic-energy for the PEs, as described in ref 1. Notably, the 11th-harmonic (16.4 eV photon energy, ℏω) PE spectra contained significant signal contributions from the 9th and 13th harmonics, which were removed by subtracting identical, tailored background spectra from both the neat water and PhOH_(aq)_ spectra (see Figure SI-2 for details).

Soft-X-ray measurements of the PhOH_(aq)_ C 1s PE intensity map and high-resolution C 1s PE spectra were performed at the beamline P04 at the PETRA III synchrotron facility, DESY (Hamburg, Germany)^23^ using our state-of-the-art LJ-PES setup *EASI* (Electronic structure from Aqueous Solutions and Interfaces).^24^ The setup is equipped with a near-ambient-pressure hemispherical electron analyzer (Scienta-Omicron HiPP-3). μ-Metal shielding ensures magnetic-field-free conditions around the interaction region, where the X-ray beam crosses the LJ, with both propagating in the horizontal (floor) plane and perpendicular to each other. In all the soft-X-ray-ionization experiments, a bias voltage of −64 V was applied to the LJ to expose the low-energy tail (LET) and spectral cutoff features. The latter was used for calibration of the KE scales in Figure 2 (main text) and SI-3 (Supporting Information); see ref 1 for further details. Differential pumping stages ensured sufficiently low pressures in both the spectrometer and the beamline. During the experiments, the average pressure inside the soft-X-ray–LJ interaction chamber was kept at ∼5 × 10^–4^ mbar by two TMPs with a combined pumping speed of ∼2600 L/s for N_2_ and three liquid-nitrogen-cooled traps with a total pumping speed of ∼35000 L/s for water. The LJ was frozen and collected by one of these traps at a distance of ∼60 cm from the LJ-injection point. The electrons were detected at an angle of 130° with respect to the light propagation direction (backward-detection configuration) and normal to the LJ, which was situated ∼0.8 mm away from the 0.8 mm-diameter entrance-skimmer orifice of the spectrometer.

The analyzer pass energy for the core-level C 1s PE map and individual PE spectra was 50 eV, and the entrance slit of the hemisphere was set to 0.1 mm, yielding a theoretical analyzer KE resolution of ∼13 meV; in practice, the resolution is ∼20–30 meV due to small stray fields within the chamber under the implemented experimental conditions. The KE step size was 125 meV for the PE map and 25 meV for the high-resolution spectra. The undulator at the beamline P04 provides circularly polarized light in the range of 250–3000 eV. We implemented photon energies in the range of 280–320 eV, which were selected by a 1200 lines/mm laminar grating. The vertical exit slit of the beamline was set to 30 μm, yielding a beam focus of 180 μm (horizontal) × 20 μm (vertical), maximizing the spatial overlap with the LJ, and beamline energy resolutions of ∼20 meV. Total experimental energy resolutions of 30–40 meV were correspondingly achieved, enabling quantitative and accurate determinations of electron KEs, BEs, peak widths, and inelastic-scattering-induced PE peak distortions. The photon-energy step size for the map data was 250 meV. The map data were normalized to the beamline photon flux, which was separately measured using a photodiode (SXUV100, Optodiode Corp.) that was introduced into the photon beam path after the last beamline optic and just before the differential pumping section between the beamline and electron spectrometer.

## Results and Discussion

### Valence Spectra

From Figure 1, we contrast the valence PE spectra of nearly neat water and a 50 mM PhOH_(aq)_ solution, applying discrete photon energies ranging from ∼10.6 eV to ∼38.1 eV using the HHG laser setup described above. The LJ-PES spectra are displayed on a KE axis, referenced to the low-energy spectral cutoff, *E*_cut_, i.e., the steep signal intensity rise at low-KE, which identifies KE = 0 eV and corresponds to the spectral onset where PEs have just enough energy to cross the solution–vacuum interface.^1^ Near *E*_cut_, scattered electrons accumulate to form the LET, characteristic of condensed-phase PES.^1,25^ The present work focuses on the LET spectral region, which – at the low photon energies considered here – is associated with quasi-elastic scattering and dominates the LJ-PES spectra. At the highest ℏω considered in Figure 1 and for both solutions, all three water outer-valence PE bands – denoted 1b_2_, 3a_1_, and 1b_1_ – are well resolved atop the broad scattering-background signal and sufficiently separated from the LET. Moving toward lower photon energies, we see that the aqueous-phase PE features with KEs > 10–14 eV are energetically separated enough from the LET to be discernible and only minorly distorted; see, for example, the water 1b_1_ bands measured at ℏω = 23.5 and 25.8 eV and shown in Figure 1, which are both already somewhat low in KE to extract accurate BEs for this band.^1^ However, for lower KEs, the liquid-water PE peaks become increasingly inseparable from the LET and distorted, making the precise determination of the peak positions elusive. At even lower ℏω values, we see that the water 1b_1_ band becomes almost indistinguishable from the LET below ∼6 eV KE, consistent with our previous studies.^1,8^ The results presented here show that the water-valence PE features behave similarly for both liquid water and the PhOH_(aq)_ solution; in the latter, slightly higher signals are observed across the spectrum due to the higher-BE PhOH_(aq)_ features, a scattering background signal from low-BE PhOH_(aq)_ features, and additional solvent-PE scattering by the interfacial PhOH_(aq)_ solute.

**Figure 1:**
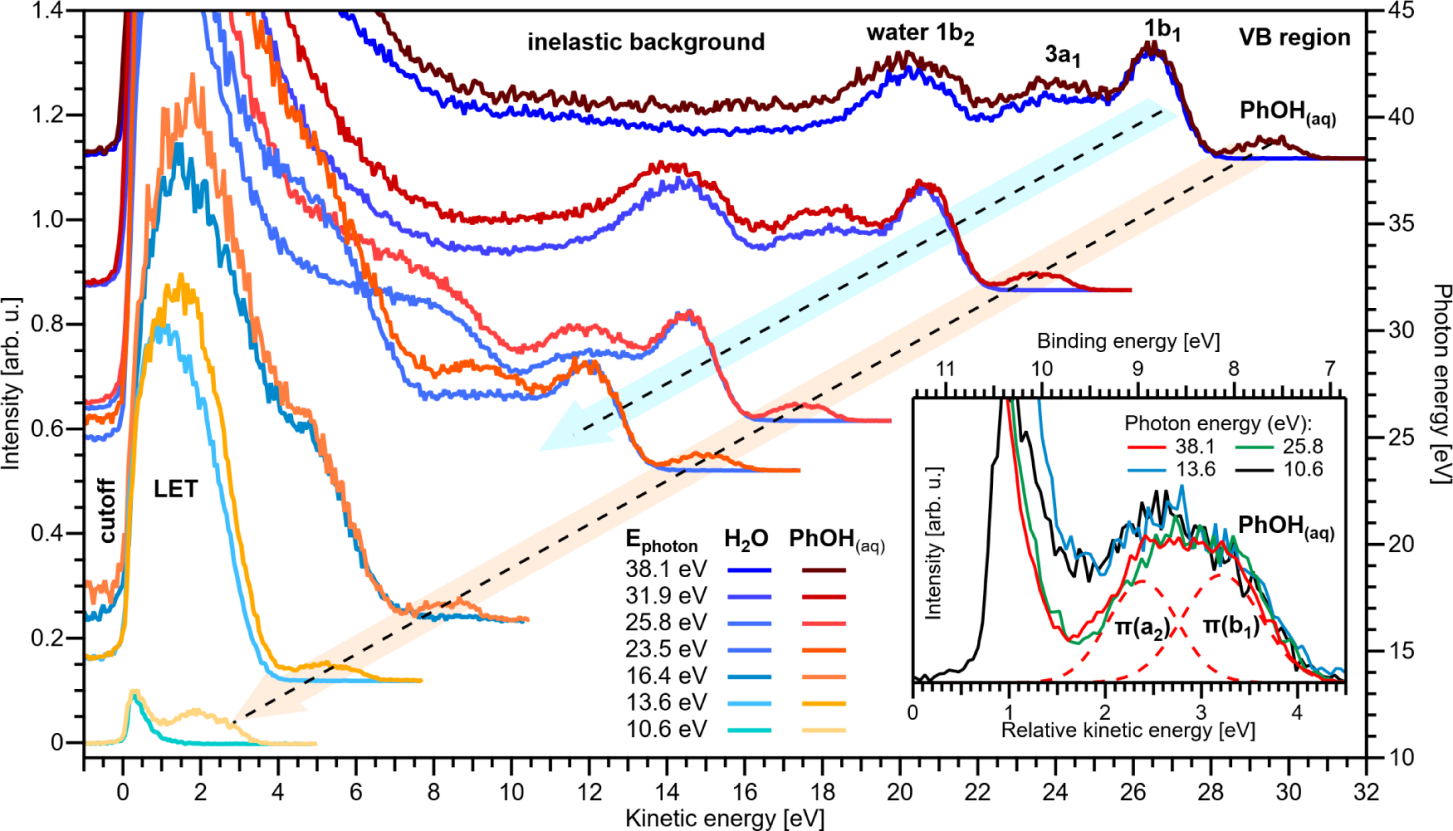
Comparison of cutoff-calibrated valence PE spectra of liquid water (dark blue to light blue colors) with 50 mM PhOH_(aq)_ (dark red to yellow) at select photon energies (right axis; errors are ± 0.1 eV) on a cutoff-calibrated KE scale (bottom axis). The peak intensities are scaled to the water 1b_1_ band, or, in its absence, to coarsely overlap the LET features. Inset: expanded view of the valence PE spectra for selected photon energies. On the lower KE scale, the PE spectra are aligned to the liquid-water 1b_1_^–1^-band position at 0 eV relative KE. As the 10.6 eV data are recorded below the vertical ionization energy of the 1b_1_^–1^-band, this spectrum is only aligned on the upper BE scale.

In contrast to the water-solvent PE features, the doublet of PE peaks associated with the two lowest-energy PhOH_(aq)_ ionizing transitions – corresponding to HOMO or π­(b_1_) and HOMO–1 or π­(a_2_) electron ejection^26^ – is observable as a distinct feature down to ∼2 eV KE, i.e., much lower than for water; see the ℏω = 10.6 eV spectrum in Figure 1. For the highest-ℏω PhOH_(aq)_ spectrum shown in Figure 1, fits to the cutoff and outer-valence band spectral features (PE KEs of ∼30 eV) yield the HOMO and HOMO–1 BEs of 8.2 ± 0.1 eV and 9.0 ± 0.1 eV, respectively, in general agreement with higher-energy-resolution 50 mM average-concentration EUV^18,27^ and our soft X-ray (8.0 ± 0.1 eV and 8.8 ± 0.1 eV) LJ data, and consistent with measurements at other solute concentrations^17,18,21,27,28^ (see the SI for further details). Minor spectral distortions of the PhOH_(aq)_ outer-valence features appear at and below ℏω = 13.6 eV (∼5 eV KE, see the inset), where the peak splitting seems to be preserved, but the HOMO–1 peak is ∼1.8 times larger than the HOMO peak. The latter may be either associated with inelastic-electron-scattering and/or near-threshold cationic resonance effects.

Most importantly, Figure 1 reveals the expected significantly reduced LKEL for valence ionization of surface-active PhOH_(aq)_ molecules compared to liquid water. The phenol-solute valence signals are well resolved down to ∼2 eV KEs (cf. 6–10 eV KE for water), with such low PhOH_(aq)_-solution LKELs implying that low-KE PEs escape the solution surface with significantly reduced inelastic scattering. Furthermore, the highest-KE PhOH_(aq)_ PE features are produced without appreciable background signals in all of the valence spectra. Additionally, for the lowest-ℏω (10.6 eV) water and solution spectra (∼2 eV KE PhOH_(aq)_-outer-valence-signal peaks), the data suggest that the LET features arise almost entirely from water-photoemission processes, i.e., PEs associated with the low-BE tail of the liquid-water 1b_1_^–1^ state.

### C 1s Core-Level Spectra

To further explore surface-active-solute PE scattering behavior, we measured high-energy-resolution C 1s core-level PE spectra from identical 50 mM PhOH_(aq)_ solutions using the soft-X-ray beamline P04 at the PETRA III synchrotron-radiation source.^23^ To quantify the LKEL, potential BE shifts and peak broadenings associated with quasi-elastic electron scattering, and the electron-KE threshold for direct and accurate BE measurements in the PhOH_(aq)_ solutions, the photon energy was scanned from 281 to 320 eV in steps of 250 meV, while PE spectra were measured over an analogous KE range to the spectra shown in Figure 1 (here, −2.5 to 35 eV). The resulting electron-signal intensity, after photon-flux normalization, is displayed in Figure 2 as a false-color PE-signal map; i.e., PE intensities are projected on both ℏω (vertical) and KE (horizontal) axes; as before, the KEs are cutoff-calibrated. The C 1s signal of interest here gives rise to the continuous diagonal signal progression, which is highlighted by the red-dashed line (extracted using a fit procedure detailed below) and, in the absence of any inelastic scattering or other peak distortions, is a direct result of the relation KE = ℏω – BE. Further, a parallel lower-intensity line is observed at a 1.7 eV lower KE. The corresponding signal intensity ratio is 5:1 and is associated with five near-equivalent carbons within the phenyl ring and one carbon neighboring the hydroxy group; this is shown in the two-dimensional PES spectra overlaid in orange, which are the electron signals measured and plotted along the KE axis for two exemplary ℏω values, 304.5 and 309.5 eV. The highest ℏω (320 eV, KE ∼ 30 eV) data yields C 1s BEs of 289.6 ± 0.1 eV and 291.3 ± 0.1 eV for the phenyl and hydroxy carbons, respectively, as referenced to the spectral-cutoff feature. Both values are ∼0.65 eV lower than the corresponding gas-phase BEs,^29^ which is smaller than common gas-aqueous-solution shifts,^30^ likely due to the average partial hydration of the PhOH solute at the aqueous surface.^31^

In addition to the PE peaks, the characteristic dominant LET signal can be seen in Figure 2 at KE = 0–5 eV, which complicates the determination of the LKEL, even for the surface-active solute. Unlike the valence ionization case, the phenol C 1s signal always resides atop a background of inelastically scattered solvent PEs, even at the lowest KEs. The constant-ℏω features near 285–288 eV arise from resonant absorption, which leads to Auger electron emission; however, only the scattering background and not the Auger features themselves are detected at the low KEs measured here. These features are shown and assigned in Figure 2B, which shows the partial-electron-yield X-ray absorption spectrum (PEY-XAS) extracted from the PE map by integrating the signal intensity in the KE = 10–30 eV range, while omitting the direct PE signal of the C 1s core-level; assignment of the absorption features is made according to ref 32 : ring C 1s → π* (285.2 ± 0.1 eV), hydroxyl C 1s → π* and ring C 1s → 3sσ (287.1 ± 0.1 eV), and hydroxyl C 1s → 3sσ (289.0 ± 0.1 eV), which, within the error bars, are the same as the gas-phase transition energies.^29,32^ The intensity minimum observed near ℏω = 297 eV originates from strong PhOH_(aq)_ absorption (e.g., by some type of shape resonance) and/or a quenched-solvent PE signal (not further discussed here).

A clear advantage of acquiring a signal map using a continuously, rather than discretely, tunable light source is that the spectral features are revealed with higher contrast and sensitivity. Even without further processing, the C 1s PE features can be identified down to very low KEs of ∼3 eV, apparently accompanied by small C 1s BE changes (as further discussed in detail below). One approach to analyze the C 1s-map data would be to subtract the LET (background) signal and isolate the (broadened) C 1s peaks down to 1–2 eV KEs; see the inset in Figure 2A. However, since a subtraction may introduce artifacts, we refrained from using such processed data for further analysis. Instead, we circumvent this issue by “slicing” the map vertically, i.e., into electron KE steps. Since the LET signal varies only slightly as a function of ℏω, this approach separates the C 1s doublet from the LET signals, projecting out the C 1s signals on an essentially ℏω-invariant background down to very low KEs, e.g., see the white (vertical) spectrum in Figure 2A, obtained by integrating the signal around a constant KE of 5 eV in a 0.25 eV range.

Each C 1s vertical data slice was masked to separate out a 10 eV-energy region of interest around the C 1s PE features (to exclude Auger resonances and LET-signal variations) and subsequently fitted with a Gaussian doublet, yielding C 1s-peak KE positions, as tracked by the red-dashed line in Figure 2; the results above ∼4 eV KEs are invariant with the choice of the masking region within a reasonable range of 6–15 eV. The dashed line tracks the position of the ring carbons and is representative for both features. The peak-height ratio and splitting were constrained to 1:5 and 1.7 eV, respectively, as determined from a fit to a high-KE, ℏω = 320 eV spectrum. The fit reveals that the C 1s features can be detected down to <5 eV KE with high accuracy. Below ∼4 eV, the fits become increasingly challenging due to overlap with the large and structured signal background (associated with Auger emission processes).

The average C 1s-KE-peak position shown in Figure 2A and marked by the red-dashed diagonal line is expected to increase with ℏω (plotted along the *y*-axis in Figure 2A) according to KE=ℏω−BE . Thus, at every ℏω value and in the absence of any additional scattering-related (or alternatively generated, see below) peak shifts, subtracting ℏω from the average PE peak position should yield a constant. The subsequent addition of the average of the C 1s BEs – 289.6 ± 0.1 eV, as determined from the high-KE data described above – should yield 0 eV, with any deviation of this sum from zero defining the absolute C 1s-KE-peak shift, ΔKE. The results of such summations are shown in Figure 3A, where ΔKE is plotted as a function of ℏω (upper) and the average PE KE (lower) on the *x*-axes, with the *y*-axis scale defined by ΔKE=KE⁡+⁡BE⁡−⁡ℏω ; negative ΔKE values indicate deviations toward smaller KE. We also measured high-resolution PE spectra at select ℏω values (see Figure SI-3 for a representative overview) to confirm the observed trend. Here, the LET signal had to be accounted for in our analysis, which made this approach more challenging and increased the error bars. For the high-resolution PE spectra fits, the LET shape was approximated by a smoothed version of the spectrum recorded at the highest photon energy, where the C 1s peaks were masked by a linear function, connecting the background on both sides of the peaks. All spectra were then fitted in a second step with a combination of this predetermined LET shape, additional broad Gaussian peaks to accommodate slight variations in the LET as a function of ℏω, and two Gaussians for the C 1s peaks (see Figure SI-3 for a representative example). The resulting peak positions (blue dots in Figure 3) are in excellent agreement with the values extracted from the C 1s map data (Figure 2A) down to electron KEs of ∼5 eV, where it becomes increasingly difficult to reliably extract peak positions from the map or regular PE data at lower energies.

**Figure 2:**
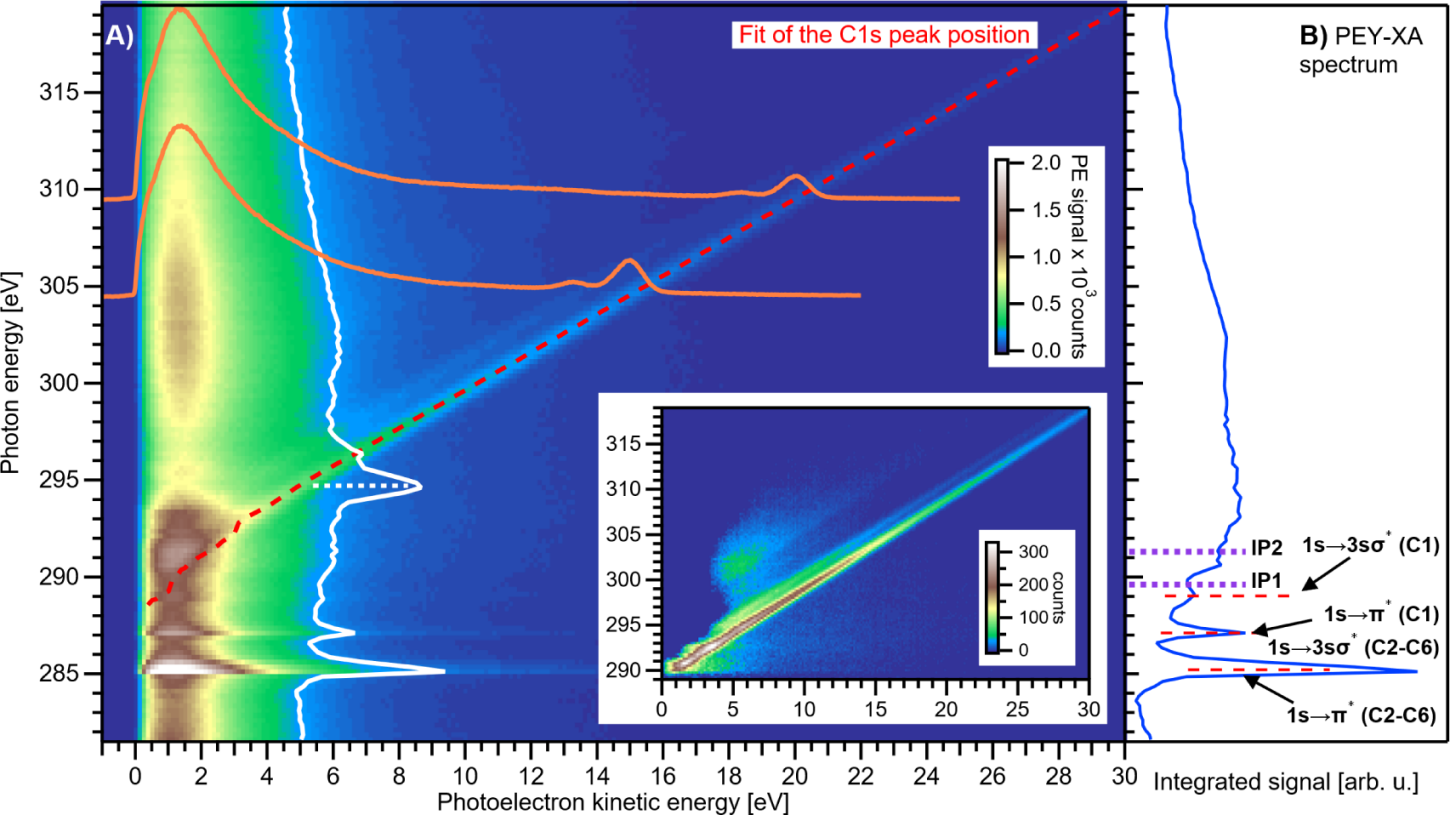
A) False-color map of cutoff-calibrated PE spectra as a function of photon energy (left axis) scanned around the C 1s ionization potentials (IPs) of 50 mM PhOH_(aq)_. Exemplary constant-photon-energy (304.5 and 309.5 eV, orange) and fixed-electron-kinetic-energy (5 eV, white) spectra are overlaid. Inset: the same map after subtraction of the Auger and LET signals. B) Partial-electron-yield X-ray-absorption (PEY-XA) spectrum obtained by summing the intensity of the 10–30 eV KE region while omitting (subtracting) the signals from the C 1s PE peaks. Resonant excitation features are labeled according to ref 32. The purple dashed lines indicate the C 1s ionization potentials of the phenyl ring (289.6 ± 0.1 eV) and hydroxyl site (291.3 ± 0.1 eV), respectively.

**Figure 3:**
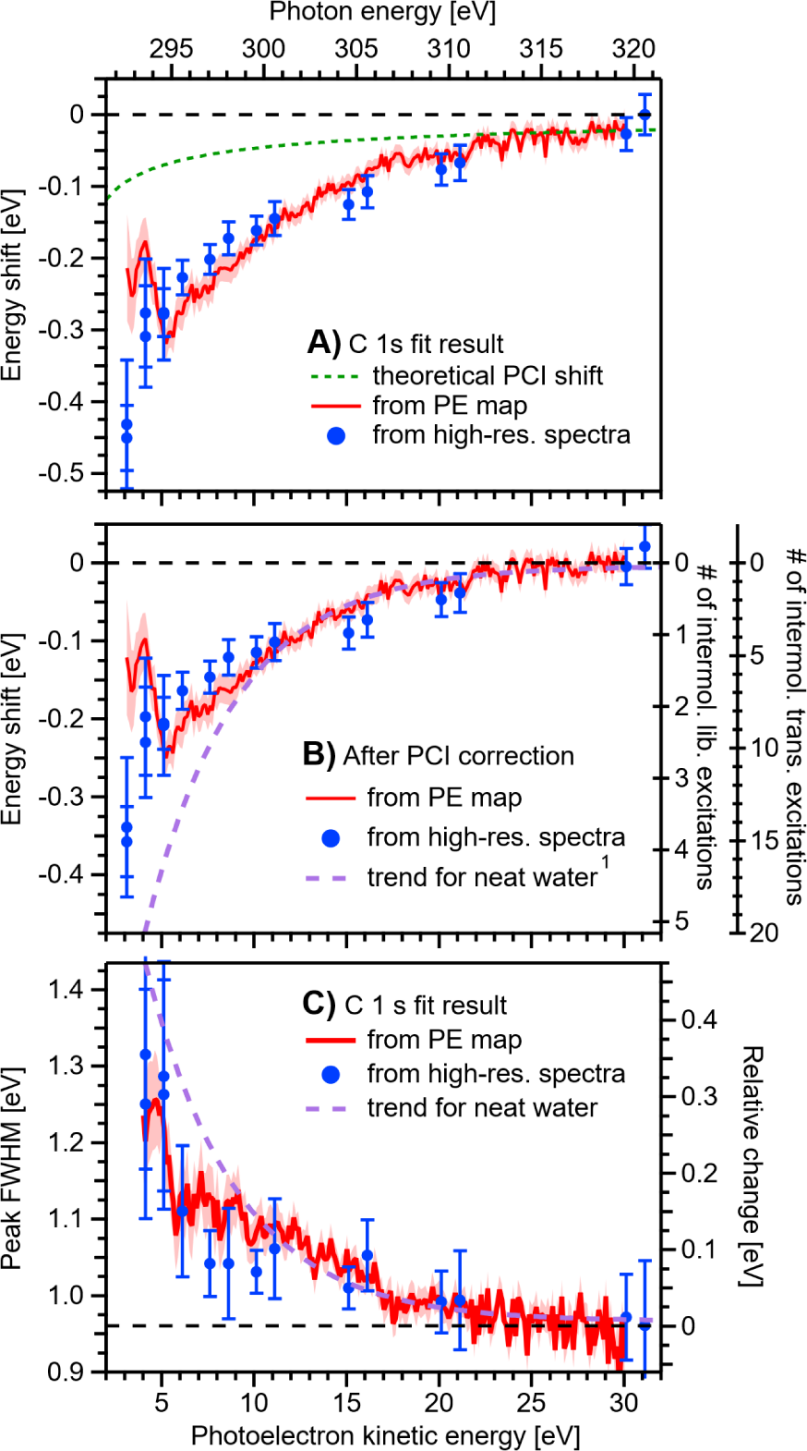
PhOH_(aq)_ C 1s-doublet average PE-peak position shift, panels A and B, and broadening, panel C, as a function of KE. Results extracted from the fit to the PE map shown in Figure 2A (highlighted by the red-dashed line) and separately measured high-resolution PE spectra (blue dots; c.f. the PE spectra in Figure SI-3). Both results agree well. A) Peak-position results, as extracted. B) As panel A but after correction for PCI effects; the green dotted line in panel A shows the expected PCI shift, which was subtracted here. The right axes show the estimated number of scattering events for intermolecular librational and translational excitations – energy losses of ∼90 and ∼25 meV, respectively – which have the highest scattering cross-sections in amorphous ice;^40^ cf. Figure SI-1. The purple dashed line shows the associated energy-shift trend for the HOMO 1b_1_ ionization channel of liquid water from ref 1. C) As panel A but showing the single-peak variation of the C 1s FWHM as a function of KE both on an absolute (only for PhOH_(aq)_; left axis) and a relative (to the average value at ≥30 eV KEs; right axis) energy scale. The purple dashed line is plotted with respect to the relative (right) scale and shows the peak-broadening trend for the neat-water 1b_1_ ionization channel, as extracted from previously reported data^1,8^ and that shown in Figure 1. Related C 1s peak area results are shown in Figure SI-5. The shaded areas and error bars throughout Figure 3 indicate the confidence intervals resulting from quadratic addition of one-σ fit errors and the overall uncertainty of the measurement.

From Figure 3A, we observe a clear decrease from the nominally expected PE KE ( ΔKE <0) as a function of ℏω and, hence, PE KE, hinting at elevated low-energy scattering contributions toward lower KEs. However, before we can quantify this effect, we must consider potential contributions from postcollision interaction (PCI), a near-threshold core-level ionization effect that leads to a low-KE asymmetry and small shifts of the core-level primary PE peaks toward lower KE.^33–36^ Briefly, secondary Auger electrons, emitted following core-ionization, eventually “overtake” the slow primary C 1s PEs and undergo Coulombic energy exchange in the field of the emerging doubly ionized ion. In effect, the primary PE – directly measured here – is retarded in the ion’s field. Here, we use a simple atomic PCI theory^33,34^ with a C 1s lifetime-broadening factor of Γ ∼ 0.1 eV (a typical value for carbon-containing molecules)^37,38^ and an Auger energy of KE_Auger_ ∼ 260 eV (again, a typical value for this kind of benzene derivative;^39^ the exact value is unimportant) to approximate the PE kinetic-energy shift associated with the PCI effect, ΔKE_PCI_:

$$\Delta {\mathrm{KE}}_{\mathrm{PCI}}=\frac{\Gamma }{2}\left(\frac{1}{\sqrt{2\mathrm{KE}}}-\frac{1}{\sqrt{2{\mathrm{KE}}_{\mathrm{Auger}}}}\right)$$

We do not expect any influence on the PCI effect from the solvent because of the PhOH_(aq)_ surface propensity. The resulting PCI curve is displayed as a green dashed line in Figure 3A, indicating an ∼25% PCI contribution to the total relative energy shift, ΔKE, near the ionization threshold. Subtraction of the PCI contribution yields the results shown in Figure 3B, thus quantifying the peak-shift contribution from quasi-elastic electron scattering alone. Surprisingly, scattering-based peak shifts emerge at ≲20 eV KEs and reach generally measurable values of 50 meV at ∼17 eV KEs, i.e., at strikingly similar values to our neat-water and homogeneously distributed aqueous solute findings.^1,8^

Further analysis of the fits to the C 1s data map shown in Figure 2A reveals a similar trend in the KE dependence of the individual surface-active-solute peak *widths* as for the associated PE KE shifts; the single-peak C 1s PE peak widths at full-width half-maximum (FWHM) are plotted versus electron KE in Figure 3C (red curve). Consistent results are extracted from the high-resolution C 1s spectra (see Figure SI-3), although these peak widths are extracted with larger uncertainties, especially at PE KEs below ∼7 eV. Generally, measurable PE peak broadenings emerge at KEs of ≲20 eV, with increasingly significant peak broadenings observed at lower KEs. The common onsets of the PE-peak position shifts and broadenings shown in Figure 3 mark the transition from predominant electronic to predominant vibrational scattering in the solvent (see Figure SI-1). Notably, however, the magnitudes of the KE shifts and peak broadenings observed for the surface-active-solute features at low KEs (<10 eV) are generally significantly lower than those observed in the previously studied cases of homogeneously distributed aqueous solutes and liquid water,^1,8^ as further discussed below.

Gas- and solid-phase water electron-scattering cross-section data are shown in Figure SI-1A,B, respectively, which are reasonable proxies for scattering processes at the liquid–gas interface and in bulk liquid water^8^ in the absence of available, reliable liquid-phase data. One possible explanation for the observed trends in Figure 3 is that a significant fraction of the PhOH_(aq)_ PEs are back and/or tangentially scattered from surrounding water molecules, thereby experiencing the same single-collision quasi-elastic (meV) scattering losses as in the case of neat water or homogeneously distributed aqueous solutions. The surface-active-solute scattering-induced KE losses and PE FWHM peak widths gradually increase to ∼200 meV as the PE KE is reduced to ∼5 eV; see the red curves in Figure 3B,C, respectively, with the magnitudes of these peak shifts and broadenings covering the energetic ranges associated with intermolecular librations and translations and intramolecular vibrations in liquid water.^9^ The librational and translational (ice) and vibrational stretching and bending modes (gas) have the highest cross sections below ∼14 eV KEs (see Figure SI-1). Thus, we may translate the measured average PE KE shifts, PE peak broadenings, and associated inelastic KE losses into average numbers of inelastic librational, translational, and vibrational scattering events experienced by the PEs produced from PhOH_(aq)_. Down to ∼5 eV KEs, this number is between one and eight for all four considered inelastic scattering modes. For example, focusing on the dominant amorphous-ice scattering channels – which have been suggested to best represent the case of liquid water^3^ – the maximum numbers of intermolecular librational and translational PhOH_(aq)_-solute-PE inelastic scattering events are indicated by the right, vertical axes in Figure 3B. Here, the observed ℏω- and electron-KE-dependent C 1s-peak energy shifts were translated into an average number of scattering events by dividing them by the average energy losses associated with the intermolecular librational and translational excitations of ∼90 and ∼25 meV, respectively;^40^ note that the resulting values are only rough estimates of the numbers of scattering events since the associated energy losses per scattering event have a rather large spread of 25–40 meV.

Based on the analysis above, we contrast the KE-dependent inelastic-scattering KE losses and the number of scattering events experienced by the surface-active-solute PEs with those occurring for similar-KE PEs generated from homogeneously distributed aqueous solutes or the water solvent; an exemplary trend for this behavior – associated with the lowest-energy ionizing transition of liquid water, 1b_1_^–1^, and our previous work^1^ – is shown in Figure 3B as a purple dashed line. We find a clear deviation in behavior between the surface-active solute and water solvent PE peak behaviors below ∼10 eV, with larger peak shifts occurring for the PE peaks associated with the homogeneously distributed aqueous-phase species (in this case, liquid water) compared to the surface-active solute. For the exemplary surface-active 50 mM PhOH_(aq)_ solute case explored here, average ∼200 meV energy losses and 1–8 electron-scattering events were inferred to occur at a PE KE of ∼5 eV. Considering our previously reported low-PE-KE liquid-water and 3 M NaCl_(aq)_ results,^1,8^ similar data analyses to those presented here allow us to estimate average 400 ± 120 meV PE KE losses – see the dashed purple curve in Figure 2B – and the occurrence of 1–20 inelastic scattering events at KEs of ∼5 eV, i.e., at the LKEL for the homogeneously distributed solution components. As shown in Figure 3C, like the PE-peak KE shifts, ∼200 meV PE peak broadenings are observed for the surface-active solute PEs at KEs of ∼5 eV (see the red curve), with 400 ± 120 meV FWHM peak broadenings extracted from homogeneously distributed aqueous solvent data at the same ∼5 eV PE KE (the purple dashed curve in Figure 3C is referenced to the right-hand “Relative change” axis and shows the average peak-broadening behavior for liquid water as a function of electron KE). Compared to the surface-active aqueous-phase solute PE features, the larger average KE losses and broader scattering-event distributions experienced by PEs produced from homogeneously distributed solution components lead to large PE-peak position extraction uncertainties, width increases, and area reductions at and below ∼5 eV KEs. At even lower PE KEs of ≲4 eV (e.g., see the ℏω = 10.7 and 13.3 eV results in Figure 1), the surface-active-solute PE features can still be readily resolved, whereas there is a complete loss of discernible PE-peak structure for the homogeneously distributed solution components. Thus, at ≲5 eV electron KEs, this indicates that much larger – and thus far experimentally unquantifiable – scattering-induced KE losses, peak-broadening extents, and average numbers of inelastic scattering events occur for PE peaks generated from liquid water and/or homogeneously distributed aqueous solutes in comparison to surface-active aqueous solutes.

## Conclusions

We have quantified the effects of low-energy-loss inelastic scattering on 1–30 eV KE electrons generated from an exemplary surface-active aqueous solute and compared them to similar results for homogeneously distributed aqueous solution components. Thereby, we have shown that for either surface-active or homogeneously distributed aqueous solution components, a similar ≳17 eV PE KE threshold occurs for the direct and accurate determination of aqueous-phase BEs, i.e., where aqueous-phase PE spectra can be measured without detectable quasi-elastic-scattering-based PE peak distortions. We attribute this threshold to increasingly significant solute-PE vibrationally inelastic scattering effects from surrounding water molecules, particularly below the crossover from predominant electronic (multiple-eV KE-loss) to vibrational (few-tens-of-meV KE-loss) scattering at 12–15 eV electron KEs. The observation that this threshold pervades with surface-active solutes, and associated partial interfacial hydration, implies that EUV or soft X-ray photons are generally required to directly and accurately measure nascent electron BEs from aqueous-phase species. By performing LJ-PES measurements as a function of photon energy below the ∼17 eV KE threshold for direct and accurate BE measurements and in the vicinity of valence and core-level ionization potentials, we have shown that moderately distorted and shifted PE peaks can be directly detected from aqueous solutions of surface-active solutes down to ∼2 eV KEs, as demonstrated using the exemplary PhOH_(aq)_ surface-active solute. In contrast, similar measurements with liquid water and homogeneously distributed aqueous solutes highlight greater PE-peak distortions and average PE KE losses at equivalent electron KEs, with associated PE-peak structures ultimately becoming indistinguishable from electron scattering-background signals at KEs ≲5 eV.^1,8^

The LJ-PES results reported here suggest that, on average, the surface-active-solute PEs undergo a lower number of low-electron-KE inelastic scattering events before they escape into vacuum compared to equivalent-KE PEs produced from homogeneously distributed aqueous-solution components. These surface-active-solute PE behaviors are expected to be useful in several important application areas, particularly when measurements of accurate electron BEs are of secondary importance. First, these results suggest that optical-pump-UV-probe time-resolved LJ-PES measurements can isolate moderately distorted PE features from surface-active solutes with ≲8 eV BEs, even when relatively low, readily accessible pump- and probe-photon energies are implemented, e.g., ℏω_pump_ ∼ 4–6 eV and ℏω_probe_ ∼ 6 eV. For example, considering the PhOH_(aq)_ surface-active solute and its ∼8 eV first ionization potential,^18^ our results suggest that the initial photoexcited-state population dynamics in this solute should be trackable at just 10–12 eV total (i.e., ℏω_pump_ + ℏω_probe_) photoexcitation energies. Second, LJ PE circular dichroism measurements have thus far been limited to KEs ≳8 eV due to PE scattering effects and related challenges in accurately determining PE-peak areas and asymmetries.^41,42^ However, the results presented here suggest that such measurements should be more readily performable with surface-active solutes and their associated lower PE-peak-detection LKELs. As PE circular dichroism effects^43,44^ are generally largest within 10 eV of the ionization thresholds of chiral species of interest, the possibility to resolve surface-active-solute PE peaks atop electron scattering backgrounds at significantly lower electron KEs will enable explorations of aqueous-phase chiral potentials over broader and more relevant energetic ranges, which is also expected to lead to measurements of larger liquid-phase chiral asymmetries.

More generally, low-KE, liquid-phase scattering mechanisms continue to be debated,^3–6^ with the data reported here expected to make an important contribution to this ongoing discussion. The surface-active-solute and solvent results described and quantified here, respectively, represent lower and upper limiting cases for average degrees of low-KE PE inelastic scattering from water molecules in aqueous solutions prior to electron escape into vacuum. Furthermore, as the surface-active-solute LJ-PES measurements were performed with surface coverages of just 0.33–0.42, the partially hydrated solute-PE source was principally confined to an interfacial monolayer. Thus, the origin and initial KE distributions of the surface-active solute PEs were more tightly defined, generally resulting in fewer electron scattering events and allowing scattering distributions to be quantified with lower experimental uncertainties and lower electron KEs. Thus, such LJ-PES measurements may offer an experimental route to more precisely extract KE-dependent, low-KE electron-scattering cross sections for liquid water and correspondingly address the current lack of reliable cross-section data in this critically important energetic range.^3^ Hence, together with appropriate scattering and spectral modeling, these and subsequent studies of electron scattering effects on LJ-PES features promise to deliver further insights into electron scattering probabilities and transfer lengths in aqueous environments, which are of paramount importance for the development of improved experimental, LJ-PES-based solute depth-profiling procedures^45–48^ and a deeper understanding of aqueous-phase (bio)­chemical interactions with low-KE electrons.^49–52^

## Supplementary Material



## Data Availability

The data relevant to this study have been deposited at the following DOI: 10.5281/zenodo.14879499

## Footnotes

1 Here, “accurate BEs” refer to values that are essentially unaffected by deleterious vibrationally inelastic scattering effects, i.e., where the average magnitude of the scattering-induced PE KE loss is less than the ∼ 50 meV energy resolutions in typical LJ-PES experiments.

2 Note that different energetic thresholds likely apply for the direct and accurate measurement of electron BEs in other (nonaqueous) solvents, which will exhibit different KE-dependent electron-scattering behaviors.

3 Generally, the broad bandwidth of the EUV light source limits our ability to accurately and precisely determine valence-electron KEs, BEs, and electron scattering induced PE peak shifts, although qualitative photon-energy- and electron-KE-dependent peak distortions, widths, and intensities and signal-to-background trends can still be analyzed.

## Notes

Low-energy gas- and solid-phase electron scattering cross-sections in water, exemplary gas-phase background-subtraction procedure adopted to extract the ℏω = 16.4 eV 50 mM PhOH spectra presented in Figure 1, exemplary high-resolution C 1s core-level photoemission spectra of 50 mM PhOH, comparison of valence-band BEs for PhOH to previous studies, soft X-ray valence-band photoemission spectrum from 50 mM PhOH and associated peak fits, and C 1s photoemission peak widths extracted from the 50 mM PhOH data as a function of photon and kinetic energy (PDF)

## References

[1] Thürmer S., Malerz S., Trinter F., Hergenhahn U., Lee C., Neumark D. M., Meijer G., Winter B., Wilkinson I. (2021). Accurate Vertical
Ionization Energy and Work Function Determinations of Liquid Water
and Aqueous Solutions. Chem. Sci..

[2] Winter B., Thürmer S., Wilkinson I. (2023). Absolute Electronic Energetics and
Quantitative Work Functions of Liquids from Photoelectron Spectroscopy. Acc. Chem. Res..

[3] Signorell R. (2020). Electron Scattering
in Liquid Water and Amorphous Ice: A Striking Resemblance. Phys. Rev. Lett..

[4] Schild A., Peper M., Perry C., Rattenbacher D., Wörner H. J. (2020). Alternative Approach for the Determination of Mean
Free Paths of Electron Scattering in Liquid Water Based on Experimental
Data. J. Phys. Chem. Lett..

[5] Thürmer S., Seidel R., Faubel M., Eberhardt W., Hemminger J. C., Bradforth S. E., Winter B. (2013). Photoelectron Angular
Distributions from Liquid Water: Effects of Electron Scattering. Phys. Rev. Lett..

[6] Suzuki Y.-I., Nishizawa K., Kurahashi N., Suzuki T. (2014). Effective attenuation
length of an electron in liquid water between 10 and 600 eV. Phys. Rev. E: Stat., Nonlinear, Soft Matter Phys..

[7] White R. D., Brunger M. J., Garland N. A., Robson R. E., Ness K. F., Garcia G., de Urquijo J., Dujko S., Petrović Z. L. (2014). Electron
swarm transport in THF and water mixtures. Eur.
Phys. J. D.

[8] Malerz S., Trinter F., Hergenhahn U., Ghrist A., Ali H., Nicolas C., Saak C.-M., Richter C., Hartweg S., Nahon L., Lee C., Goy C., Neumark D. M., Meijer G., Wilkinson I., Winter B., Thürmer S. (2021). Low-energy
constraints on photoelectron spectra measured from liquid water and
aqueous solutions. Phys. Chem. Chem. Phys..

[9] Luckhaus D., Yamamoto Y. I., Suzuki T., Signorell R. (2017). Genuine binding
energy of the hydrated electron. Sci. Adv..

[10] Yamamoto Y.-I., Karashima S., Adachi S., Suzuki T. (2016). Wavelength
Dependence
of UV Photoemission from Solvated Electrons in Bulk Water, Methanol,
and Ethanol. J. Phys. Chem. A.

[11] Shreve A. T., Yen T. A., Neumark D. M. (2010). Photoelectron
spectroscopy of hydrated
electrons. Chem. Phys. Lett..

[12] Kumar G., Roy A., McMullen R. S., Kutagulla S., Bradforth S. E. (2018). The influence
of aqueous solvent on the electronic structure and non-adiabatic dynamics
of indole explored by liquid-jet photoelectron spectroscopy. Faraday Discuss..

[13] Riley J. W., Wang B., Woodhouse J. L., Assmann M., Worth G. A., Fielding H. H. (2018). Unravelling the Role of an Aqueous Environment on the
Electronic Structure and Ionization of Phenol Using Photoelectron
Spectroscopy. J. Phys. Chem. Lett..

[14] Henley A., Riley J. W., Wang B., Fielding H. H. (2020). An experimental
and computational study of the effect of aqueous solution on the multiphoton
ionisation photoelectron spectrum of phenol. Faraday Discuss..

[15] Roy A., Seidel R., Kumar G., Bradforth S. E. (2018). Exploring
Redox Properties of Aromatic Amino Acids in Water: Contrasting Single
Photon vs Resonant Multiphoton Ionization in Aqueous Solutions. J. Phys. Chem. B.

[16] Scholz M. S., Fortune W. G., Tau O., Fielding H. H. (2022). Accurate
Vertical
Ionization Energy of Water and Retrieval of True Ultraviolet Photoelectron
Spectra of Aqueous Solutions. J. Phys. Chem.
Lett..

[17] Ghosh D., Roy A., Seidel R., Winter B., Bradforth S., Krylov A. I. (2012). First-Principle Protocol for Calculating Ionization
Energies and Redox Potentials of Solvated Molecules and Ions: Theory
and Application to Aqueous Phenol and Phenolate. J. Phys. Chem. B.

[18] Richter C., Dupuy R., Trinter F., Buttersack T., Cablitz L., Gholami S., Stemer D., Nicolas C., Seidel R., Winter B., Bluhm H. (2024). Surface accumulation
and acid–base equilibrium of phenol at the liquid–vapor
interface. Phys. Chem. Chem. Phys..

[19] Rao Y., Subir M., McArthur E. A., Turro N. J., Eisenthal K. B. (2009). Organic
ions at the air/water interface. Chem. Phys.
Lett..

[20] Kurahashi N., Karashima S., Tang Y., Horio T., Abulimiti B., Suzuki Y.-I., Ogi Y., Oura M., Suzuki T. (2014). Photoelectron
spectroscopy of aqueous solutions: Streaming potentials of NaX (X
= Cl, Br, and I) solutions and electron binding energies of liquid
water and X^–^. J. Chem. Phys..

[21] Heitland J., Lee J. C., Ban L., Abma G. L., Fortune W. G., Fielding H. H., Yoder B. L., Signorell R. (2024). Valence Electronic
Structure of Interfacial Phenol in Water Droplets. J. Phys. Chem. A.

[22] Metje J., Borgwardt M., Moguilevski A., Kothe A., Engel N., Wilke M., Al-Obaidi R., Tolksdorf D., Firsov A., Brzhezinskaya M., Erko A., Kiyan I. Y., Aziz E. F. (2014). Monochromatization
of femtosecond XUV light pulses
with the use of reflection zone plates. Opt.
Express..

[23] Viefhaus J., Scholz F., Deinert S., Glaser L., Ilchen M., Seltmann J., Walter P., Siewert F. (2013). The Variable Polarization
XUV Beamline P04 at PETRA III: Optics, mechanics and their performance. Nucl. Instrum. Methods Phys. Res., Sect. A.

[24] Malerz S., Haak H., Trinter F., Stephansen A. B., Kolbeck C., Pohl M., Hergenhahn U., Meijer G., Winter B. (2022). A setup for studies of photoelectron
circular dichroism from chiral molecules in aqueous solution. Rev. Sci. Instrum..

[25] Wilson C.
D., Dukes C. A., Baragiola R. A. (2001). Search for the plasmon in condensed
water. Phys. Rev. B.

[26] Debies T. P., Rabalais J. W. (1972). Photoelectron spectra of substituted
benzenes. J. Electron Spectrosc. Relat. Phenom..

[27] Yamamoto Y.-I., Hirano T., Ishiyama T., Morita A., Suzuki T. (2025). Gas–Liquid
Interface of Aqueous Solutions of Surface Active Aromatic Molecules
Studied Using Extreme Ultraviolet Laser Photoelectron Spectroscopy
and Molecular Dynamics Simulation. J. Am. Chem.
Soc..

[28] Lin P.-C., Wu Z.-H., Chen M.-S., Li Y.-L., Chen W.-R., Huang T.-P., Lee Y.-Y., Wang C. C. (2017). Interfacial Solvation
and Surface pH of Phenol and Dihydroxybenzene Aqueous Nanoaerosols
Unveiled by Aerosol VUV Photoelectron Spectroscopy. J. Phys. Chem. B.

[29] Hill A., Sa’adeh H., Cameron D., Wang F., Trofimov A. B., Larionova E. Y., Richter R., Prince K. C. (2021). Positional
and Conformational
Isomerism in Hydroxybenzoic Acid: A Core-Level Study and Comparison
with Phenol and Benzoic Acid. J. Phys. Chem.
A.

[30] Winter B., Weber R., Hertel I. V., Faubel M., Jungwirth P., Brown E. C., Bradforth S. E. (2005). Electron binding energies of aqueous
alkali and halide ions: EUV photoelectron spectroscopy of liquid solutions
and combined ab initio and molecular dynamics calculations. J. Am. Chem. Soc..

[31] Kusaka R., Ishiyama T., Nihonyanagi S., Morita A., Tahara T. (2018). Structure
at the air/water interface in the presence of phenol:A study using
heterodyne-detected vibrational sum frequency generation and molecular
dynamics simulation. Phys. Chem. Chem. Phys..

[32] Lin Y.-S., Lu K.-T., Lee Y. T., Tseng C.-M., Ni C.-K., Liu C.-L. (2014). Near-Edge X-ray
Absorption Fine Structure Spectra and
Site-Selective Dissociation of Phenol. J. Phys.
Chem. A.

[33] Kuchiev M.
U., Sheĭnerman S. A. (1989). Post-collision
interaction in atomic processes. Sov. Phys.
Usp..

[34] van
der Straten P., Morgenstern R., Niehaus A. (1988). Angular Dependent Post-Collision
Interaction in Auger Processes. Z. Phys. D:
At., Mol. Clusters.

[35] Lindblad A., Fink R. F., Bergersen H., Lundwall M., Rander T., Feifel R., Öhrwall G., Tchaplyguine M., Hergenhahn U., Svensson S. (2005). Postcollision interaction
in noble gas clusters: Observation of differences in surface and bulk
line shapes. J. Chem. Phys..

[36] Kassühlke B., Romberg R., Averkamp P., Feulner P. (1998). Substrate Mediated
Suppression of Postcollision Interaction Effects. Phys. Rev. Lett..

[37] Hergenhahn U. (2004). Vibrational
structure in inner shell photoionization of molecules. J. Phys. B: At., Mol. Opt. Phys..

[38] Campbell J. L., Papp T. (2001). Widths of the Atomic
K–N7 Levels. At.
Data Nucl. Data Tables.

[39] Siegbahn, K. ; Nordling, C. ; Johansson, G. ; Hedman, J. ; Hedén, P. F. ; Hamrin, K. ; Gelius, U. ; Bergmark, T. ; Werme, L. O. ; Manne, R. ESCA: Applied to Free Molecules; North-Holland Publishing Company, 1969.

[40] Michaud M., Wen A., Sanche L. (2003). Cross Sections
for Low-Energy (1–100 eV) Electron
Elastic and Inelastic Scattering in Amorphous Ice. Radiat. Res..

[41] Pohl M. N., Malerz S., Trinter F., Lee C., Kolbeck C., Wilkinson I., Thürmer S., Neumark D. M., Nahon L., Powis I., Meijer G., Winter B., Hergenhahn U. (2022). Photoelectron
circular dichroism in angle-resolved photoemission from liquid fenchone. Phys. Chem. Chem. Phys..

[42] Stemer D., Thürmer S., Trinter F., Hergenhahn U., Pugini M., Credidio B., Malerz S., Wilkinson I., Nahon L., Meijer G., Powis I., Winter B. (2025). Photoelectron
circular dichroism of aqueous-phase alanine. Chem. Sci..

[43] Janssen M. H., Powis I. (2014). Detecting chirality
in molecules by imaging photoelectron circular
dichroism. Phys. Chem. Chem. Phys..

[44] Sparling C., Townsend D. (2025). Two decades of imaging
photoelectron circular dichroism:
From first principles to future perspectives. Phys. Chem. Chem. Phys..

[45] Holmberg S., Moberg R., Yuan Z. C., Siegbahn H. (1986). Angle resolved electron
spectroscopy for measurement of surface segregation phenomena in liquids
and solutions. J. Electron Spectrosc. Relat.
Phenom..

[46] Björneholm O., Werner J., Ottosson N., Öhrwall G., Ekholm V., Winter B., Unger I., Söderström J. (2014). Deeper Insight
into Depth-Profiling of Aqueous Solutions Using Photoelectron Spectroscopy. J. Phys. Chem. C.

[47] Dupuy R., Filser J., Richter C., Buttersack T., Trinter F., Gholami S., Seidel R., Nicolas C., Bozek J., Egger D., Oberhofer H., Thürmer S., Hergenhahn U., Reuter K., Winter B., Bluhm H. (2023). Ångstrom-Depth Resolution with Chemical Specificity at the Liquid-Vapor
Interface. Phys. Rev. Lett..

[48] Gallo T., Michailoudi G., Valerio J., Adriano L., Heymann M., Schulz J., Marinho R. d. R. T., Callefo F., Walsh N., Ohrwall G. (2024). Aqueous Ammonium
Nitrate Investigated Using Photoelectron
Spectroscopy of Cylindrical and Flat Liquid Jets. J. Phys. Chem. B.

[49] Boudaiffa B., Cloutier P., Hunting D., Huels M. A., Sanche L. (2000). Resonant Formation
of DNA Strand Breaks by Low-Energy (3 to 20 eV) Electrons. Science.

[50] Wang F., Archirel P., Muroya Y., Yamashita S., Pernot P., Yin C., El Omar A. K., Schmidhammer U., Teuler J.-M., Mostafavi M. (2017). Effect of the solvation state of
electron in dissociative electron attachment reaction in aqueous solutions. Phys. Chem. Chem. Phys..

[51] Wang C.-R., Nguyen J., Lu Q.-B. (2009). Bond Breaks of Nucleotides by Dissociative
Electron Transfer of Nonequilibrium Prehydrated Electrons: A New Molecular
Mechanism for Reductive DNA Damage. J. Am. Chem.
Soc..

[52] Ma J., Wang F., Denisov S. A., Adhikary A., Mostafavi M. (2017). Reactivity
of prehydrated electrons toward nucleobases and nucleotides in aqueous
solution. Sci. Adv..

