# Supplementary material for: Low-Energy Photoelectron Spectroscopy and Scattering from Aqueous Solutions and the Role of Solute Surface Activity
Source: J Am Chem Soc. 2025 Jun 2;147(23):19868–77. doi: 10.1021/jacs.5c04263 (PMC12164337; doi:10.1021/jacs.5c04263)
Supplement: Supplementary file 1 [file ja5c04263_si_001.pdf]

Supplementary Information

Low-energy photoelectron spectroscopy and scattering in aqueous solutions and the role of solute surface activity

Stephan Thürmer[1*], Dominik Stemer[2], Florian Trinter[2, 3], Igor Yu Kiyan[4], Bernd Winter[2], and Iain Wilkinson[4*]

[1] *Department of Chemistry, Graduate School of Science, Kyoto University, Kitashirakawa-Oiwakecho, Sakyo, Kyoto 606-8501 Japan*

[2] *Molecular Physics Department, Fritz-Haber-Institut der Max-Planck-Gesellschaft, Faradayweg 4-6, 14195 Berlin, Germany*

[3] *Institut für Kernphysik, Goethe-Universität Frankfurt, Max-von-Laue-Straße 1, 60438 Frankfurt am Main*

[4] *Institute for Electronic Structure Dynamics, Helmholtz-Zentrum Berlin für Materialien und Energie, Hahn-Meitner-Platz 1, 14109 Berlin, Germany*

*Corresponding authors: thuermer@kuchem.kyoto-u.ac.jp; iain.wilkinson@helmholtz-berlin.de

Supplementary Figures

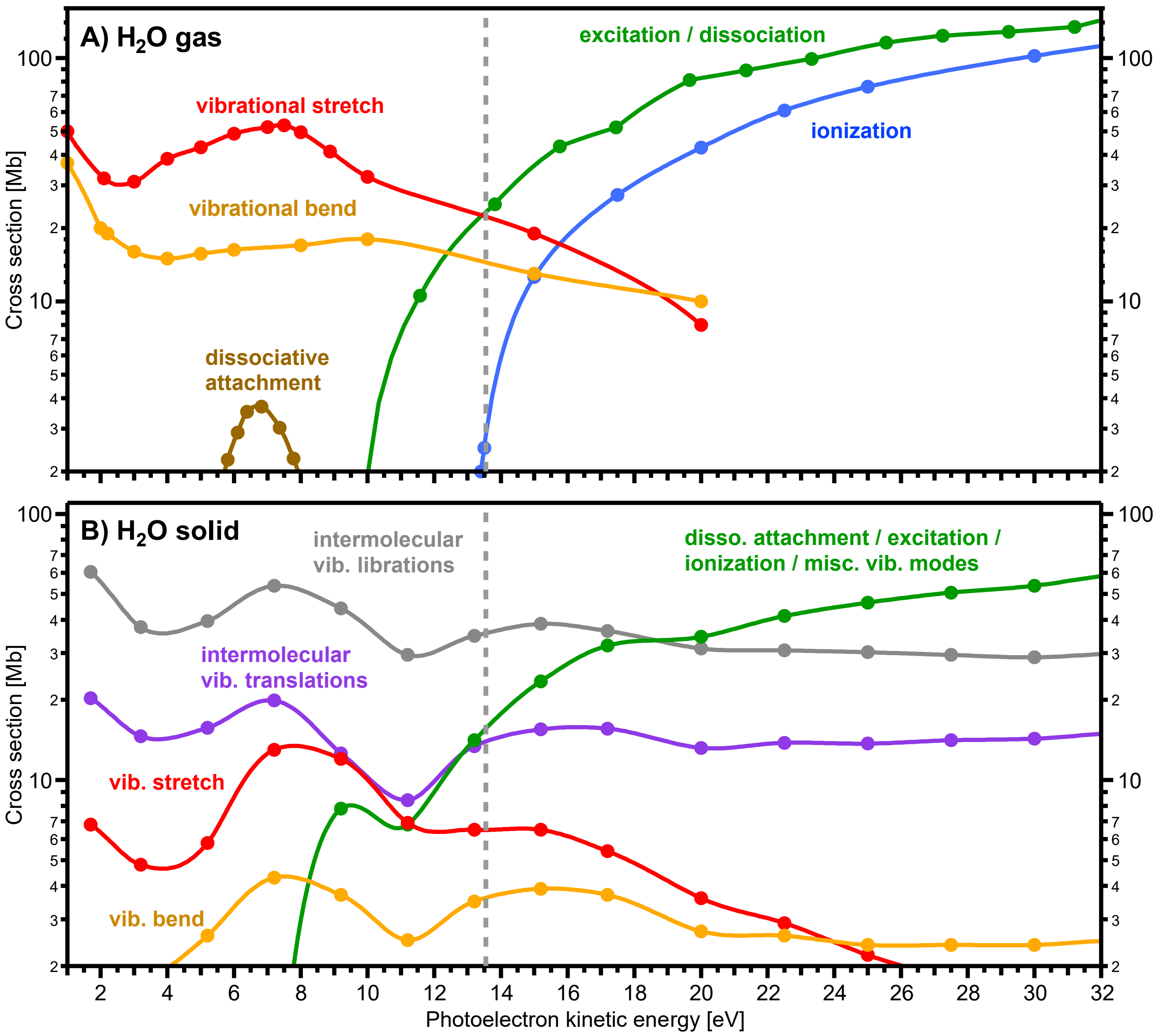

Figure SI-1: Cross sections for various channels of electron scattering with H_2O molecules. **A)** Gas phase: ionization (blue), vibrational stretch (red), and vibrational bend (yellow) from Ref. 1; direct dissociation following excitation (green) from Ref. 2; and dissociative electron attachment (brown) from Ref. 3. **B)** Solid phase: intermolecular librations (grey), intermolecular translations (purple), vibrational stretch (red), and vibrational bend (yellow) and other modes such as dissociative attachment, excitation, ionization (green). All values are from Ref. 4. The solid-phase scattering models and cross sections likely well describe the liquid-phase case considered here, although this continues to be debated.[5-8]

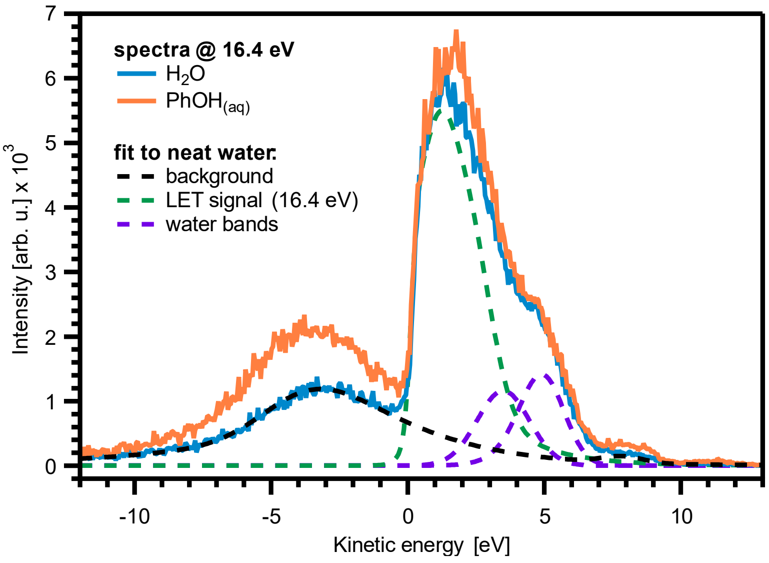

Figure SI-2: Subtraction procedure for removing contributions from gas-phase water and other harmonics from the HHG spectrum measured at 16.4 eV. First, the neat-water spectrum was fitted with a combination of Exponentially Modified Gaussians (EMG, for asymmetric contributions) and Gaussians to describe the identifiable ħω = 16.4 eV water bands (purple dashed lines), the LET contribution of the 16.4 eV PE spectrum itself (green dashed line) and the background-signal contribution from gas-phase water and the neighbouring harmonics (black dashed line). The latter was then subtracted from both the neat-water and $PhOH_{(aq)}$ spectra to yield the subtracted spectra shown in Fig. 1.

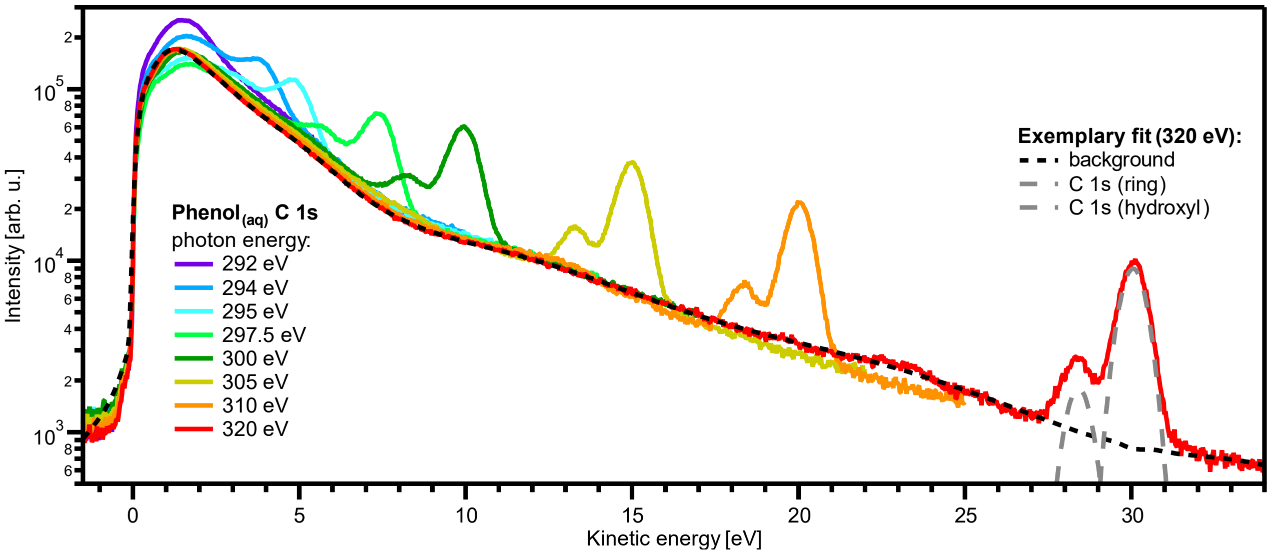

Figure SI-3: High-resolution PE spectra of 50 mM $PhOH_{(aq)}$, measured in addition to the PE map shown in Fig. 2 in the main text at various photon energies spanning 292 eV to 320 eV; a bias voltage of -64 V was applied to expose the spectral cutoff. The bottom axis shows cutoff-calibrated KEs, with the left intensity axis presented on a log scale. Notably, the C 1s doublet peak of $PhOH_{(aq)}$ is well distinguishable even below 5 eV atop the large LET feature, for example, see the blue spectrum recorded at ħω = 294 eV. An exemplary fit to the LET and the C 1s peaks is shown for the 320-eV spectrum. The LET shape is approximated by a noise-reduced version of the highest-energy spectrum, where the C 1s peak has been masked by a linear function connecting the background on both sides. All spectra were fitted in a second step with a combination of this LET shape (dotted line), additional broad Gaussian peaks to accommodate slight variations in the LET shape as a function of photon energy (not shown), and two Gaussians for the two C 1s peaks.

Comparison of valence-band BEs for $PhOH_{(aq)}$ to previous studies.

High-electron-kinetic-energy valence $PhOH_{(aq)}$ PE spectra have been reported in droplet[9-10] and liquid-jet[11-13] photoemission spectroscopy (LJ-PES) experiments, allowing electron BEs to be directly extracted from the measured spectra. $PhOH_{(aq)}$ LJ-PES measurements were first reported in Ref. 11 with high solute concentrations (0.75 M) and photon energies (200 eV), respectively revealing 7.8 ± 0.1 and 8.6 ± 0.1 eV HOMO and HOMO-1 BEs (referenced to the liquid-water $1b_1$ valence BE in the concentrated solution, assuming a similar $1b_1$ IE as detected in neat water). Recently, up to 0.5 eV BE increases were observed with decreasing solute concentration in EUV LJ-PES measurements; associated 50 mM-$PhOH_{(aq)}$-solution measurements yielded HOMO and HOMO-1 IEs of 8.0 ± 0.1 eV and 8.8 ± 0.1 eV, respectively, again referenced to the liquid-water $1b_1$ valence BE in the solutions.[12] The latter values are in agreement with the 38.1 eV-photon-energy results presented in Fig. 1 (8.2 ± 0.1 eV and 9.0 ± 0.1 eV) in the main text (see the figure inset) and our synchrotron-based results (8.0 ± 0.1 eV and 8.8 ± 0.1 eV; see Fig. SI-4) recorded at a 260 eV photon energy under similar conditions to Fig. 2A. These results were both obtained from 50 mM $PhOH_{(aq)}$ solutions, adopting a constrained 0.8 eV peak spacing, and highlight a slight reduction (<100 meV, in agreement with Yamamoto *et al.*[13]) of the water $1b_1$ BE in these solutions, specifically when they are energy referenced to the spectral cutoff. They are, however, slightly higher than the recently reported 25 mM-concentration 40 eV-photon-energy results from Yamamoto *et al.*,[13] where valence phenol binding energies of 7.90 ± 0.04 eV and 8.72 ± 0.03 eV are presented.

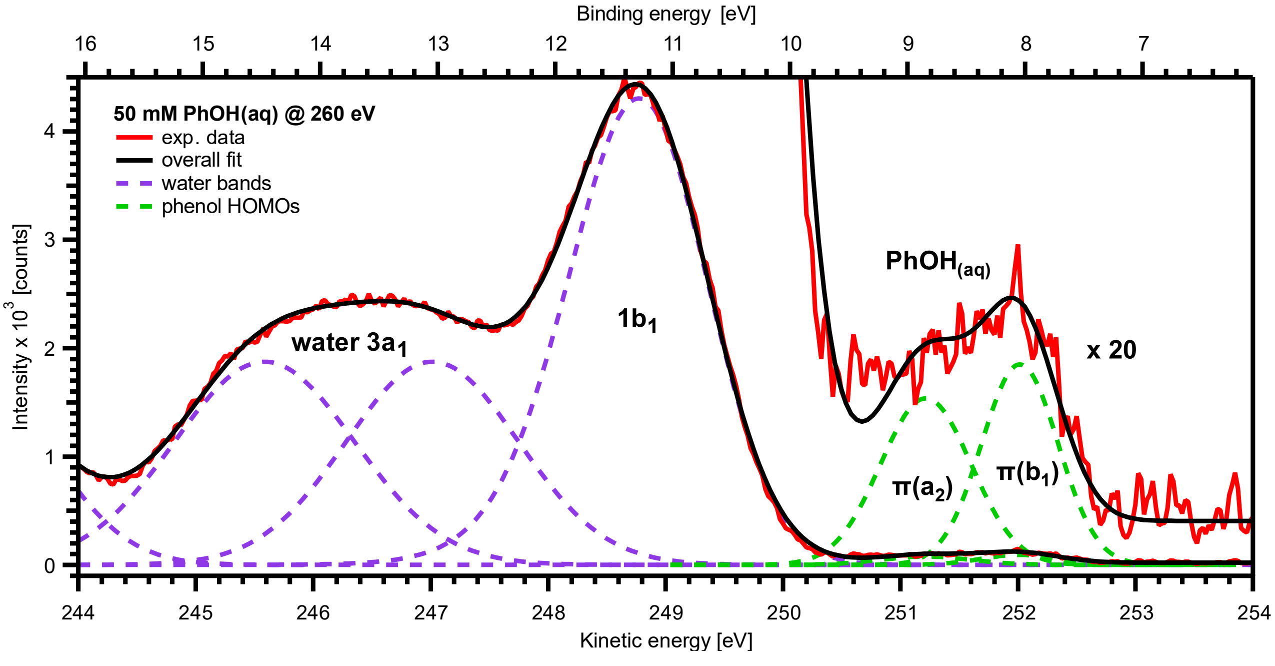

Figure SI-4: Valence-band PE spectrum of 50 mM $PhOH_{(aq)}$ on a cutoff-calibrated KE scale (bottom axis), which was measured at the PETRA III synchrotron facility using the EASI spectrometer and a calibrated 260.06 ± 0.05 eV photon energy. The top axis shows binding energies according to the relation KE = ħω – BE. The water features (purple dashed lines) in the spectrum were fitted with three Gaussians (one for the $1b_2$ band and two – constrained to the same height – for the split $3a_1$ band.[14]) and one exponentially modified Gaussian shape (for the water $1b_1$), while two Gaussians were employed for the phenol HOMO features (green dashed lines). The energetic separation of the latter was constrained to 0.8 eV in accordance with our EUV results and the 50 mM-concentration results reported in Ref. 12. To the right, the outer-valence $PhOH_{(aq)}$ ionisation features are reproduced with a 20-fold higher intensity for a clearer view on the phenol features.

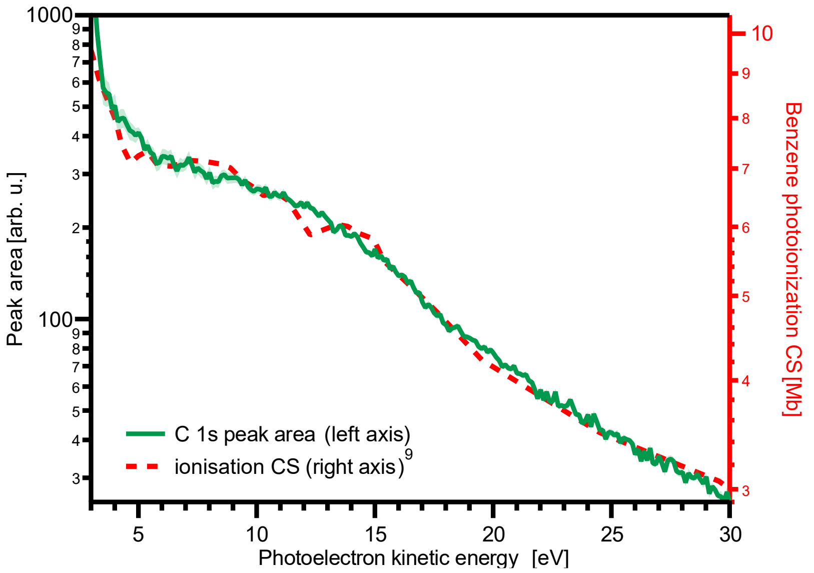

Figure SI-5: Total C 1s peak area of the Gaussian-doublet fit to the 50 mM $PhOH_{(aq)}$ PE map (Fig. 2A in the main text). The peak area increases towards lower KE, which is indicative of the increasing C 1s photoionization cross section; the cross-section values for gas-phase benzene C 1s are added for comparison (red dashed line, right axis).[15] No diminished peak signal intensities are observed below a 10-15 eV kinetic-energy threshold, unlike the case for neat water and homogeneously distributed solutes, where the signal steeply declines.[16-17]

References

1. Itikawa, Y.; Mason, N., Cross Sections for Electron Collisions with Water Molecules. *J. Phys. Chem. Ref. Data* **2005,** *34*, 1-22.
2. Harb, T.; Kedzierski, W.; McConkey, J. W., Production of ground state OH following electron impact on H_2O. *J. Chem. Phys.* **2001,** *115*, 5507-5512.
3. Melton, C. E., Cross Sections and Interpretation of Dissociative Attachment Reactions Producing OH−, O−, and H−in H_2O. *J. Chem. Phys.* **1972,** *57*, 4218-4225.
4. Michaud, M.; Wen, A.; Sanche, L., Cross Sections for Low-Energy (1–100 eV) Electron Elastic and Inelastic Scattering in Amorphous Ice. *Radiat. Res.* **2003,** *159*, 3-22.
5. Signorell, R., Electron Scattering in Liquid Water and Amorphous Ice: A Striking Resemblance. *Phys. Rev. Lett.* **2020,** *124*, 205501.
6. Schild, A.; Peper, M.; Perry, C.; Rattenbacher, D.; Wörner, H. J., Alternative Approach for the Determination of Mean Free Paths of Electron Scattering in Liquid Water Based on Experimental Data. *J. Phys. Chem. Lett.* **2020,** *11*, 1128-1134.
7. Thürmer, S.; Seidel, R.; Faubel, M.; Eberhardt, W.; Hemminger, J. C.; Bradforth, S. E.; Winter, B., Photoelectron Angular Distributions from Liquid Water: Effects of Electron Scattering. *Phys. Rev. Lett.* **2013,** *111*, 173005.
8. Suzuki, Y.-I.; Nishizawa, K.; Kurahashi, N.; Suzuki, T., Effective attenuation length of an electron in liquid water between 10 and 600 eV. *Phys. Rev. E: Stat., Nonlinear, Soft Matter Phys.* **2014,** *90*, 010302(R).
9. Lin, P.-C.; Wu, Z.-H.; Chen, M.-S.; Li, Y.-L.; Chen, W.-R.; Huang, T.-P.; Lee, Y.-Y.; Wang, C. C., Interfacial Solvation and Surface pH of Phenol and Dihydroxybenzene Aqueous Nanoaerosols Unveiled by Aerosol VUV Photoelectron Spectroscopy. *J. Phys. Chem. B* **2017,** *121*, 1054-1067.
10. Heitland, J.; Lee, J. C.; Ban, L.; Abma, G. L.; Fortune, W. G.; Fielding, H. H.; Yoder, B. L.; Signorell, R., Valence Electronic Structure of Interfacial Phenol in Water Droplets. *J. Phys. Chem. A* **2024,** *128*, 7396-7406.
11. Ghosh, D.; Roy, A.; Seidel, R.; Winter, B.; Bradforth, S.; Krylov, A. I., First-Principle Protocol for Calculating Ionization Energies and Redox Potentials of Solvated Molecules and Ions: Theory and Application to Aqueous Phenol and Phenolate. *J. Phys. Chem. B* **2012,** *116*, 7269-7280.
12. Richter, C.; Dupuy, R.; Trinter, F.; Buttersack, T.; Cablitz, L.; Gholami, S.; Stemer, D.; Nicolas, C.; Seidel, R.; Winter, B.; Bluhm, H., Surface accumulation and acid–base equilibrium of phenol at the liquid–vapor interface. *Phys. Chem. Chem. Phys.* **2024,** *26*, 27292-27300.
13. Yamamoto, Y.-i.; Hirano, T.; Ishiyama, T.; Morita, A.; Suzuki, T., Gas–Liquid Interface of Aqueous Solutions of Surface Active Aromatic Molecules Studied Using Extreme Ultraviolet Laser Photoelectron Spectroscopy and Molecular Dynamics Simulation. *J. Am. Chem. Soc.* **2025,** *147*, 4026-4037.
14. Winter, B.; Weber, R.; Widdra, W.; Dittmar, M.; Faubel, M.; Hertel, I. V., Full Valence Band Photoemission from Liquid Water Using EUV Synchrotron Radiation. *J. Phys. Chem. A* **2004,** *108*, 2625-2632.
15. Rennie, E. E.; Kempgens, B.; Köppe, H. M.; Hergenhahn, U.; Feldhaus, J.; Itchkawitz, B. S.; Kilcoyne, A. L. D.; Kivimäki, A.; Maier, K.; Piancastelli, M. N.; Polcik, M.; Rüdel, A.; Bradshaw, A. M., A comprehensive photoabsorption, photoionization, and shake-up excitation study of the C 1s cross section of benzene. *J. Chem. Phys.* **2000,** *113*, 7362-7375.
16. Thürmer, S.; Malerz, S.; Trinter, F.; Hergenhahn, U.; Lee, C.; Neumark, D. M.; Meijer, G.; Winter, B.; Wilkinson, I., Accurate Vertical Ionization Energy and Work Function Determinations of Liquid Water and Aqueous Solutions. *Chem. Sci.* **2021,** *12*, 10558-10582.
17. Malerz, S.; Trinter, F.; Hergenhahn, U.; Ghrist, A.; Ali, H.; Nicolas, C.; Saak, C.-M.; Richter, C.; Hartweg, S.; Nahon, L.; Lee, C.; Goy, C.; Neumark, D. M.; Meijer, G.; Wilkinson, I.; Winter, B.; Thürmer, S., Low-energy constraints on photoelectron spectra measured from liquid water and aqueous solutions. *Phys. Chem. Chem. Phys.* **2021,** *23*, 8246-8260.
